# Supplementary material for: LIMPACAT: Multi-omics attention transformer for immune prediction in liver cancer using whole-slide imaging
Source: PLoS One. 2026 Jan 9;21(1):e0339667. doi: 10.1371/journal.pone.0339667 (PMC12788640; doi:10.1371/journal.pone.0339667)
Supplement: S1 Fig — Positive correlations confirm the effectiveness of the filtering process. (PDF) [file pone.0339667.s001.pdf]

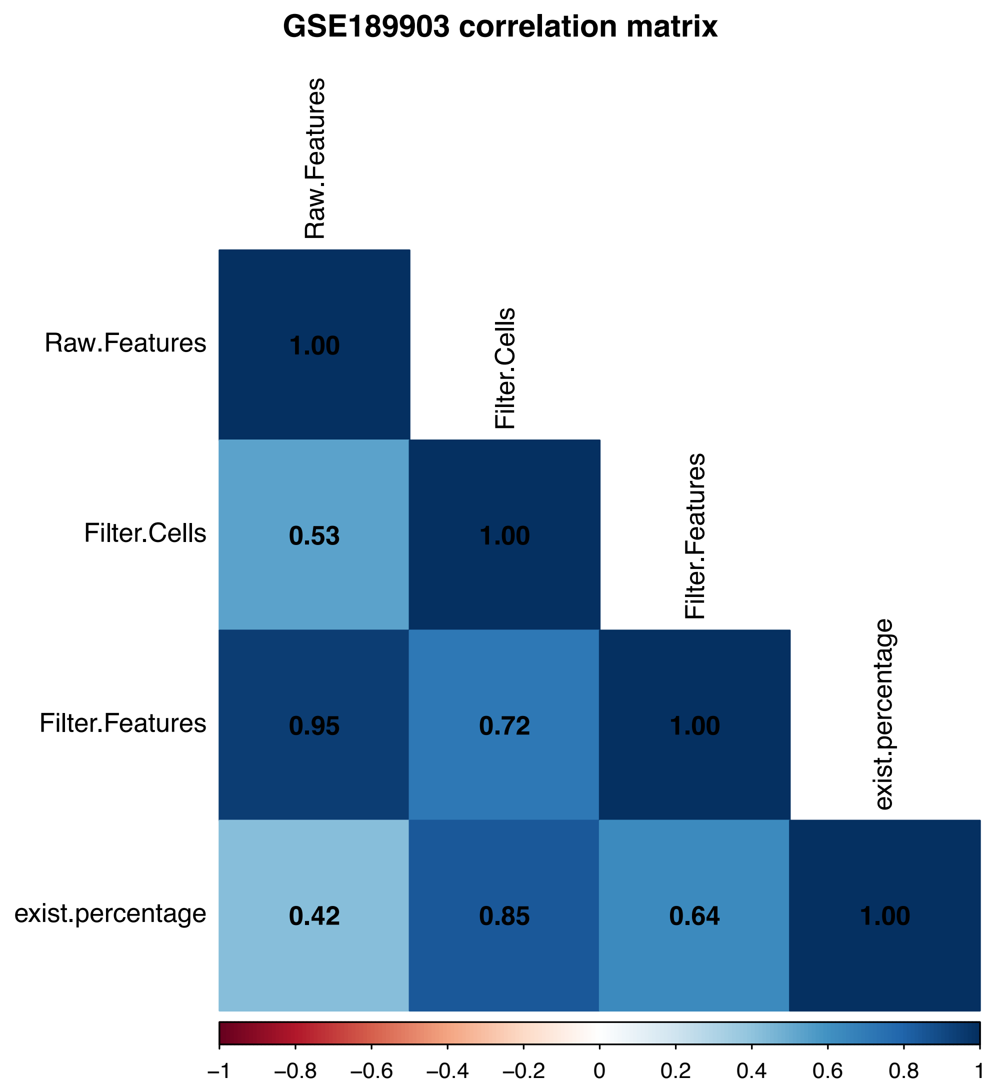

S1 Fig Correlation matrix showing relationships among filtering metrics (Raw Features, Filtered Cells, Filtered Features, Retained Cell Percentage) in the GSE189903 dataset. Positive correlations confirm the effectiveness of the filtering process.
