# Supplementary figures and images for: LIMPACAT: Multi-omics attention transformer for immune prediction in liver cancer using whole-slide imaging
Source: PLoS One. 2026 Jan 9;21(1):e0339667. doi: 10.1371/journal.pone.0339667 (PMC12788640; doi:10.1371/journal.pone.0339667)

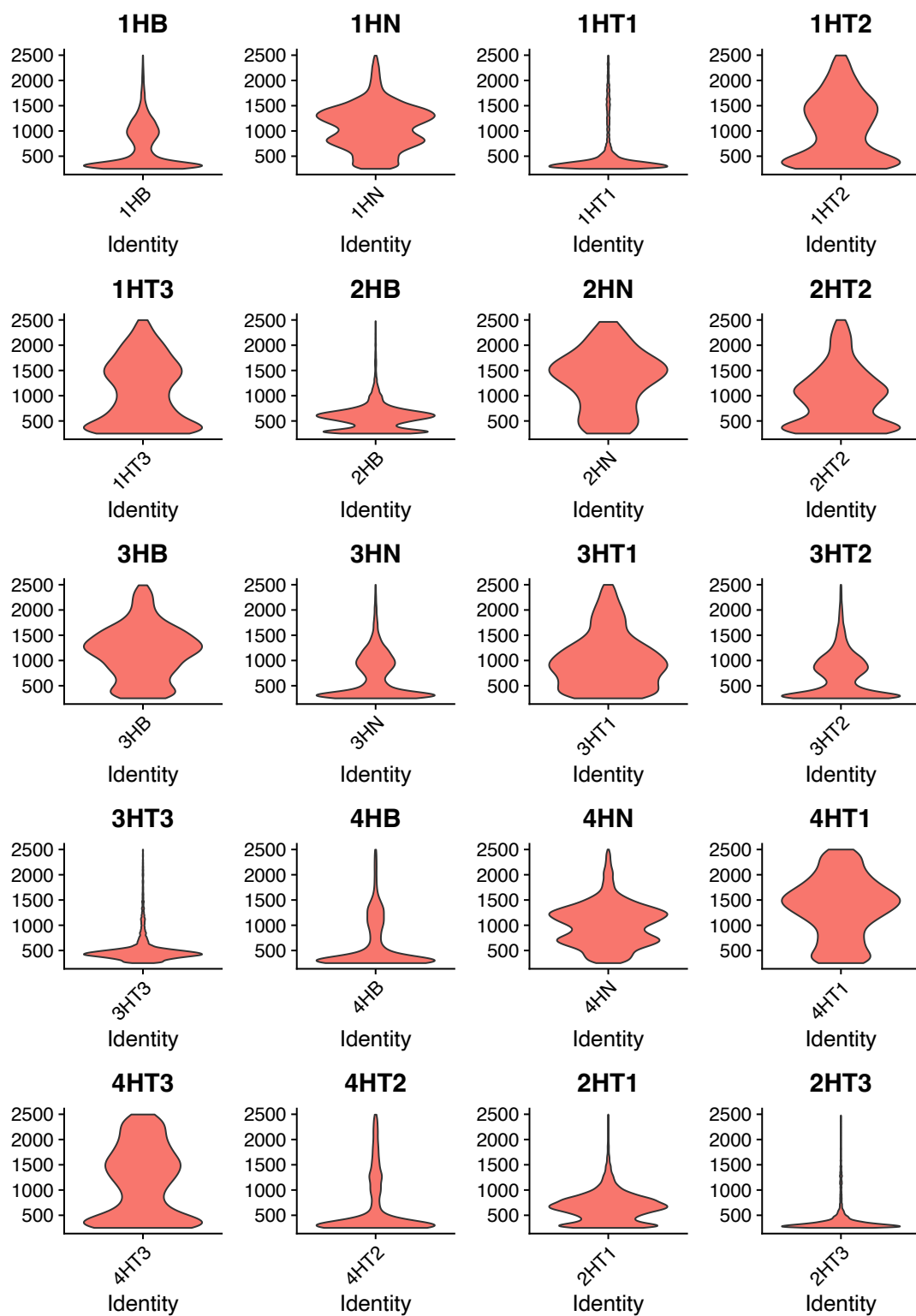

S2 Fig Filtered Cell Population with nFeature Counts Between 250 and 2500

Supplement: S2 Fig — (PDF) [file pone.0339667.s002.pdf]

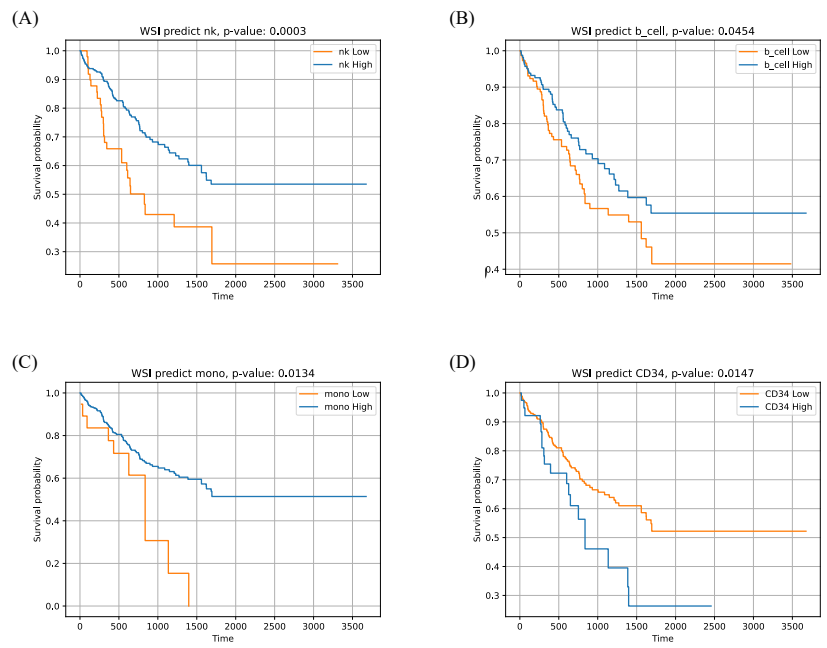

S13 Fig Survival analysis based on WSI-predicted immune levels.

Supplement: S13 Fig — (PDF) [file pone.0339667.s013.pdf]
