# Supplementary material for: LIMPACAT: Multi-omics attention transformer for immune prediction in liver cancer using whole-slide imaging
Source: PLoS One. 2026 Jan 9;21(1):e0339667. doi: 10.1371/journal.pone.0339667 (PMC12788640; doi:10.1371/journal.pone.0339667)
Supplement: S4 Fig — The weak correlation indicates that mitochondrial gene content is independent of sequencing depth, suggesting minimal impact of sequencing depth on mitochondrial percentage. (PDF) [file pone.0339667.s004.pdf]

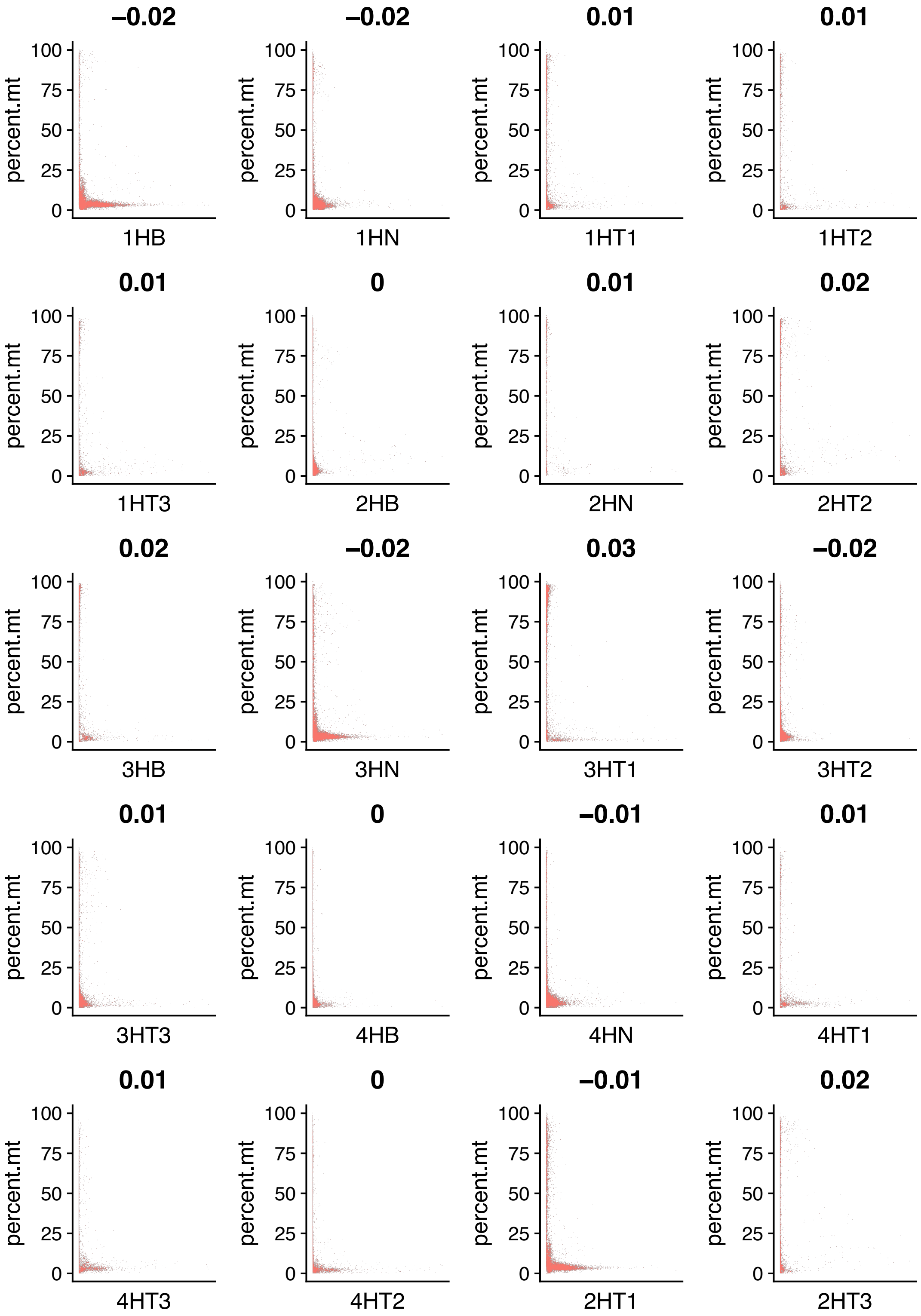

S4 Fig Correlation between nCount and mitochondrial gene percentage (mt percent) across samples. The weak correlation indicates that mitochondrial gene content is independent of sequencing depth, suggesting minimal impact of sequencing depth on mitochondrial percentage.
