# Supplementary material for: LIMPACAT: Multi-omics attention transformer for immune prediction in liver cancer using whole-slide imaging
Source: PLoS One. 2026 Jan 9;21(1):e0339667. doi: 10.1371/journal.pone.0339667 (PMC12788640; doi:10.1371/journal.pone.0339667)
Supplement: S5 Fig — The strong positive correlation confirms that cells retained after filtering have consistent sequencing depth and gene detection, supporting the reliability of high-quality data for downstream analysis. (PDF) [file pone.0339667.s005.pdf]

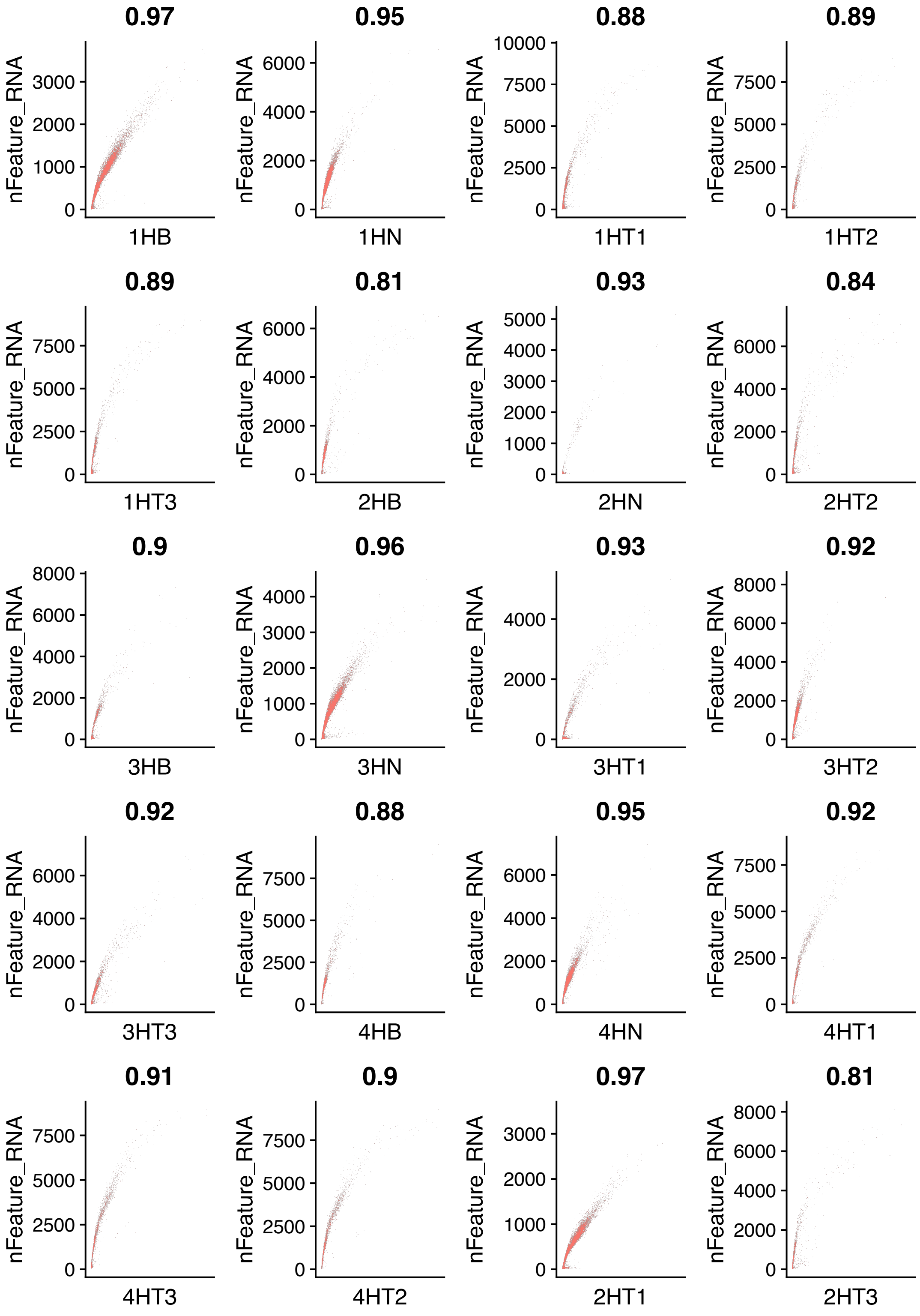

S5 Fig Correlation between nCount and nFeature across samples. The strong positive correlation confirms that cells retained after filtering have consistent sequencing depth and gene detection, supporting the reliability of high-quality data for downstream analysis.
