# Supplementary material for: LIMPACAT: Multi-omics attention transformer for immune prediction in liver cancer using whole-slide imaging
Source: PLoS One. 2026 Jan 9;21(1):e0339667. doi: 10.1371/journal.pone.0339667 (PMC12788640; doi:10.1371/journal.pone.0339667)
Supplement: S6 Fig — (A) UMAP plot showing the distribution of 28 clusters, highlighting additional cell state distinctions. (B) UMAP plot colored by sample identity, revealing the distribution of cells across clusters. Lower ARI score suggests less consistency compared to log normalization. (PDF) [file pone.0339667.s006.pdf]

CCA

(A)

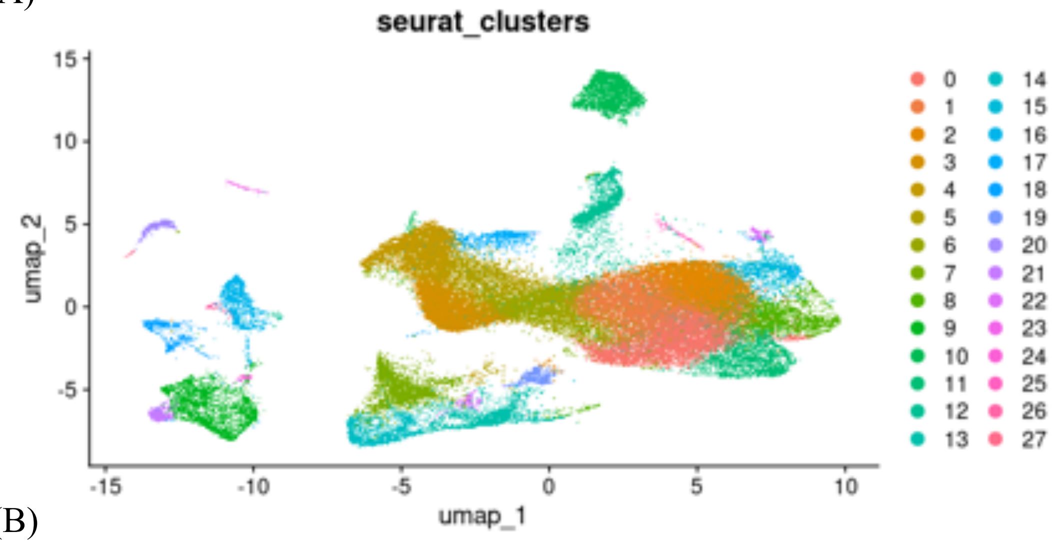

(B)

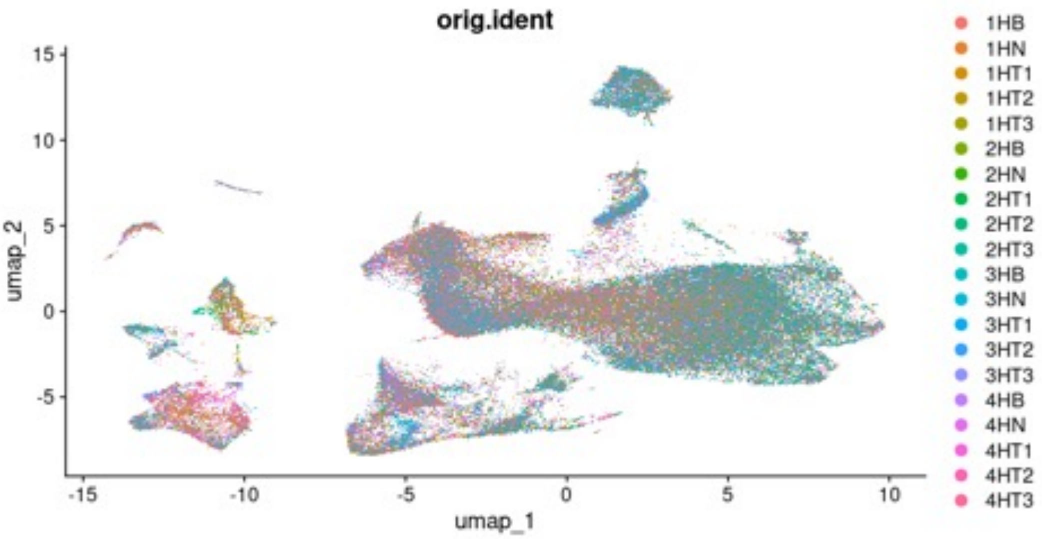

S6 Fig UMAP clustering of scRNA-seq data post-CCA normalization. (A) UMAP plot showing the distribution of 28 clusters, highlighting additional cell state distinctions. (B) UMAP plot colored by sample identity, revealing the distribution of cells across clusters. Lower ARI score suggests less consistency compared to log normalization.
