# Supplementary material for: LIMPACAT: Multi-omics attention transformer for immune prediction in liver cancer using whole-slide imaging
Source: PLoS One. 2026 Jan 9;21(1):e0339667. doi: 10.1371/journal.pone.0339667 (PMC12788640; doi:10.1371/journal.pone.0339667)
Supplement: S7 Fig — (A) UMAP plot with 23 defined clusters, indicating preserved cell type distinctions. (B) UMAP plot by sample identity, showing the spread of cells across clusters. The ARI score indicates moderate clustering consistency and successful batch effect mitigation. (PDF) [file pone.0339667.s007.pdf]

SCT

(A)

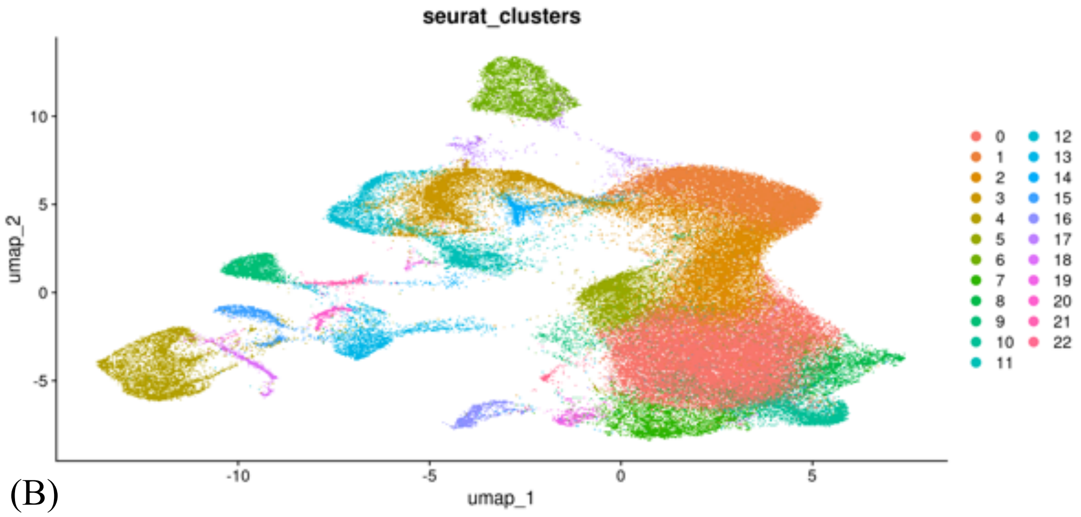

(B)

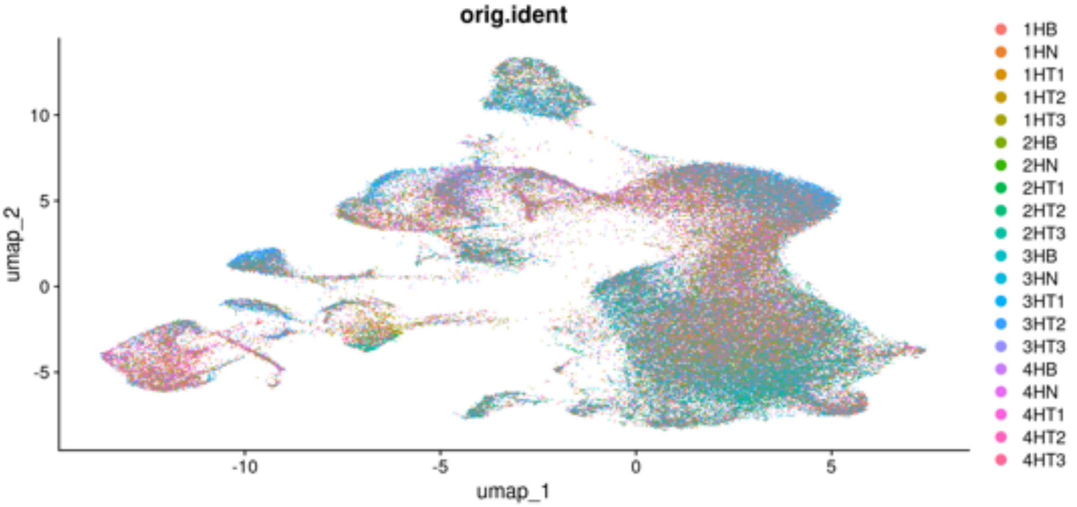

S7 Fig UMAP clustering of scRNA-seq data by sct normalization. (A) UMAP plot with 23 defined clusters, indicating preserved cell type distinctions. (B) UMAP plot by sample identity, showing the spread of cells across clusters. The ARI score indicates moderate clustering consistency and successful batch effect mitigation.
