# Supplementary material for: LIMPACAT: Multi-omics attention transformer for immune prediction in liver cancer using whole-slide imaging
Source: PLoS One. 2026 Jan 9;21(1):e0339667. doi: 10.1371/journal.pone.0339667 (PMC12788640; doi:10.1371/journal.pone.0339667)
Supplement: S8 Fig — (PDF) [file pone.0339667.s008.pdf]

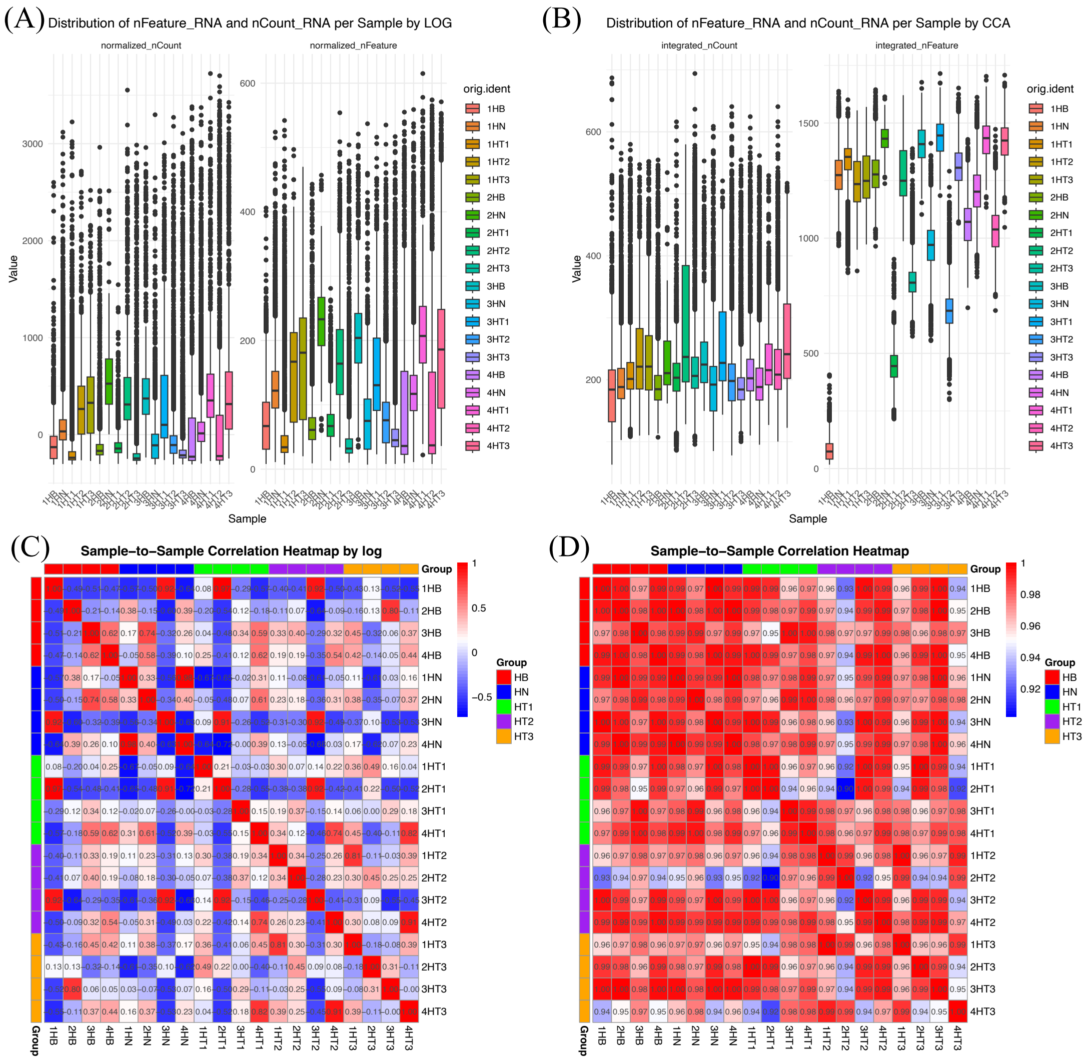

S8 Fig nFeature and nCount distributions and sample-to-sample correlations for sct normalization (A, C) and CCA (B, D). The boxplots show the distributions of nFeature and nCount across samples, and the heatmaps display sample-to-sample correlation patterns under each normalization method.
