# Supplementary material for: LIMPACAT: Multi-omics attention transformer for immune prediction in liver cancer using whole-slide imaging
Source: PLoS One. 2026 Jan 9;21(1):e0339667. doi: 10.1371/journal.pone.0339667 (PMC12788640; doi:10.1371/journal.pone.0339667)
Supplement: S9 Fig — UMAP visualizations show identified cell types, based on annotation results following each normalization method. (PDF) [file pone.0339667.s009.pdf]

(A)

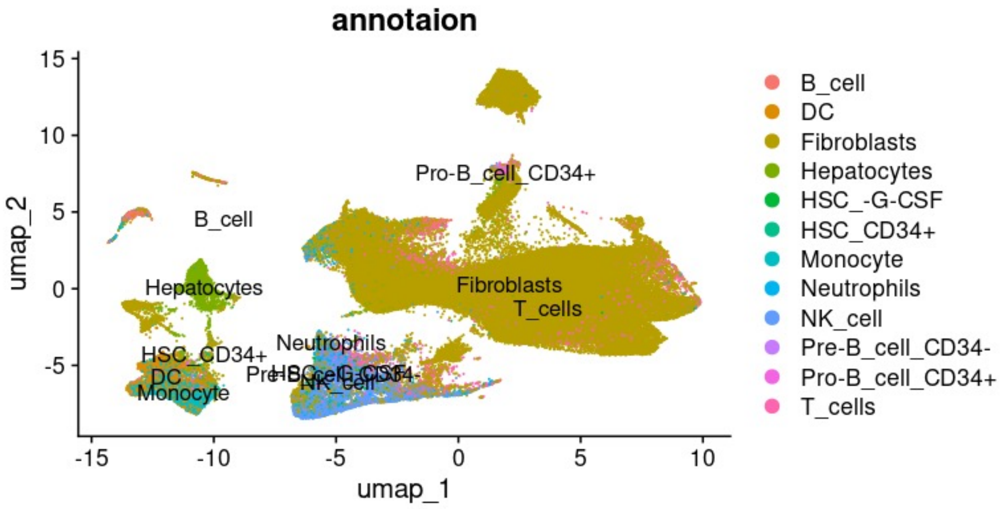

(B)

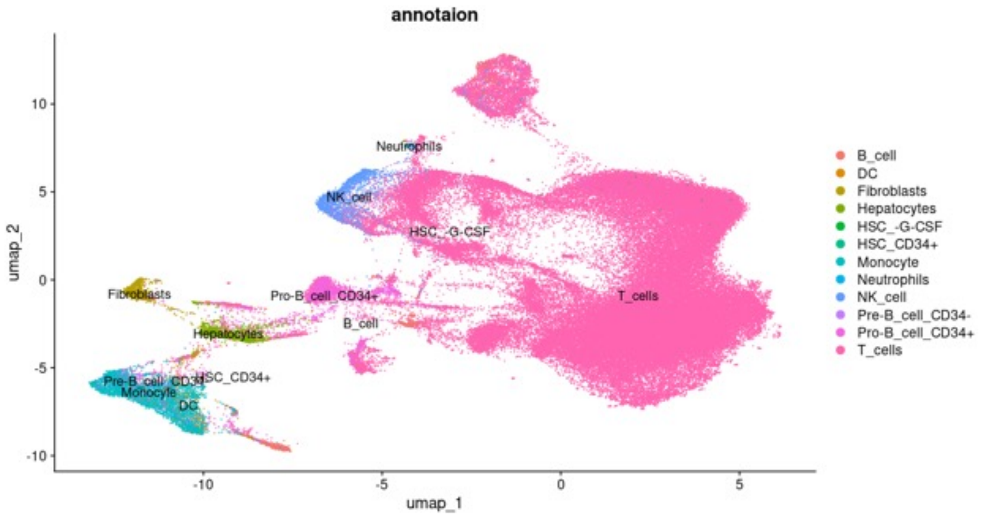

S9 Fig Cell type annotations after clustering with sct normalization (A) and CCA (B). UMAP visualizations show identified cell types, based on annotation results following each normalization method.
