# Supplementary material for: LIMPACAT: Multi-omics attention transformer for immune prediction in liver cancer using whole-slide imaging
Source: PLoS One. 2026 Jan 9;21(1):e0339667. doi: 10.1371/journal.pone.0339667 (PMC12788640; doi:10.1371/journal.pone.0339667)
Supplement: S10 Fig — and survival analysis. (A) Training and validation accuracy/error rates for the cell deconvolution model show no overfitting. (B) ARI comparison of immune cell composition predictions across normalization methods (LOG, CCA, SCT) for LIHC samples. (C) Correlation of immune cell composition and survival times across normalization methods, with CCA and SCT showing consistency. (D) Immune cell composition distributions in LIHC samples, showing similarity between CCA and SCT. (PDF) [file pone.0339667.s010.pdf]

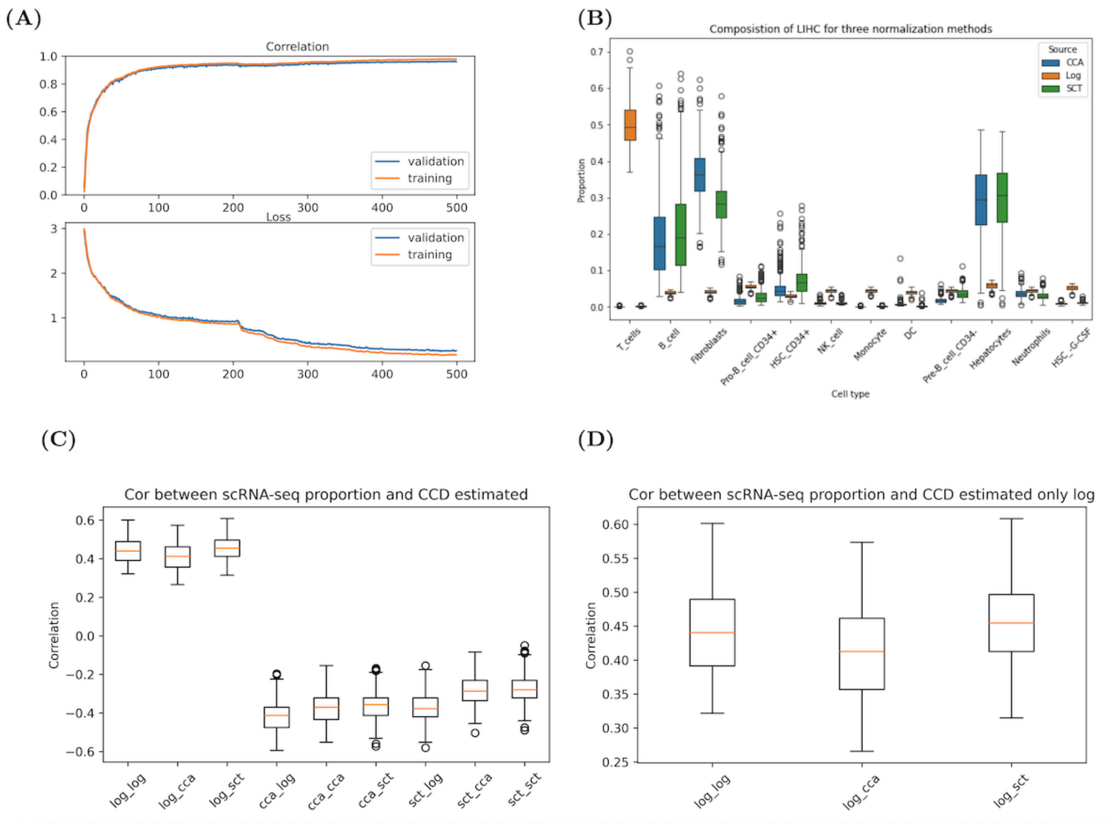

S10 Fig Summary of cell composition deconvolution model performance, normalization comparisons, and survival analysis. (A) Training and validation accuracy/error rates for the cell deconvolution model show no overfitting. (B) ARI comparison of immune cell composition predictions across normalization methods (LOG, CCA, SCT) for LIHC samples. (C) Correlation of immune cell composition and survival times across normalization methods, with CCA and SCT showing consistency. (D) Immune cell composition distributions in LIHC samples, showing similarity between CCA and SCT.
