# Supplementary material for: LIMPACAT: Multi-omics attention transformer for immune prediction in liver cancer using whole-slide imaging
Source: PLoS One. 2026 Jan 9;21(1):e0339667. doi: 10.1371/journal.pone.0339667 (PMC12788640; doi:10.1371/journal.pone.0339667)
Supplement: S11 Fig — (PDF) [file pone.0339667.s011.pdf]

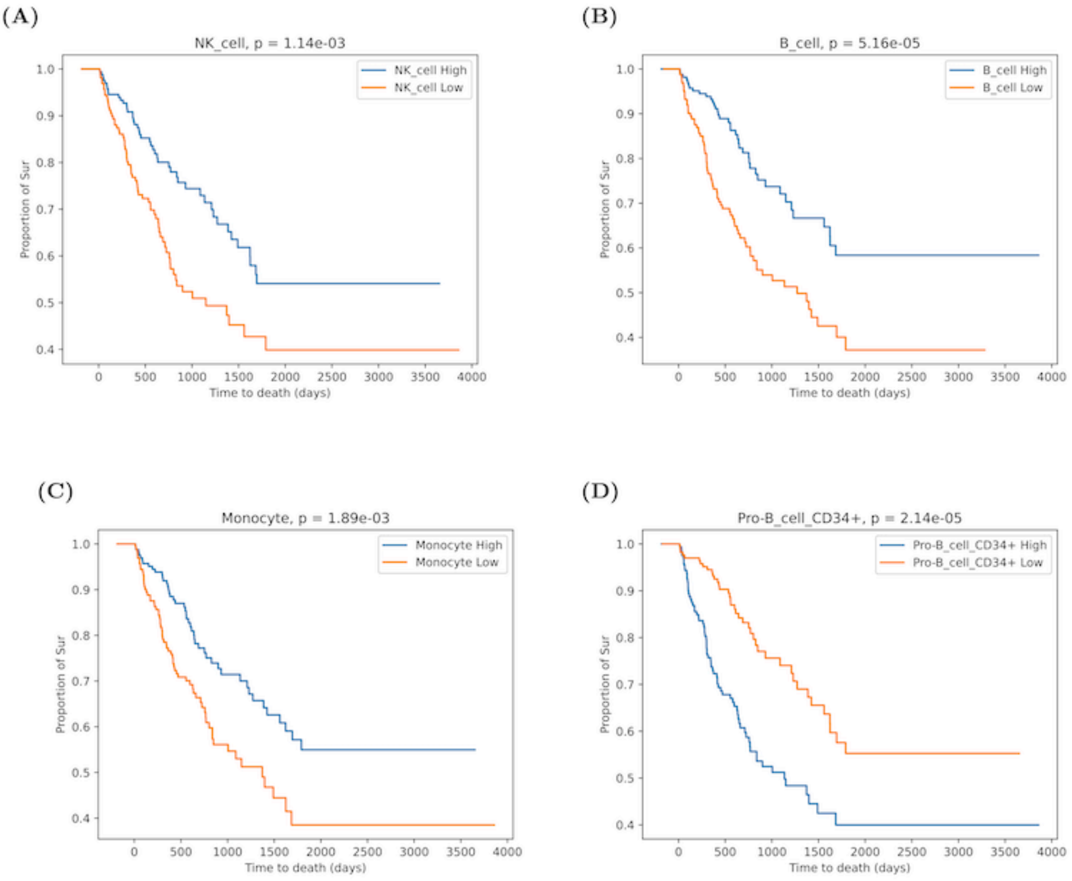

S11 Fig Survival analysis indicates a positive association between higher levels of NK cells, B cells, and monocytes with longer survival times, whereas a negative association is observed with higher levels of CD34+ B cells.
