# Supplementary material for: LIMPACAT: Multi-omics attention transformer for immune prediction in liver cancer using whole-slide imaging
Source: PLoS One. 2026 Jan 9;21(1):e0339667. doi: 10.1371/journal.pone.0339667 (PMC12788640; doi:10.1371/journal.pone.0339667)
Supplement: S12 Fig — Pearson correlation coefficients between model-inferred cell-type proportions and ground truth proportions derived from sorted PBMC populations. (PDF) [file pone.0339667.s012.pdf]

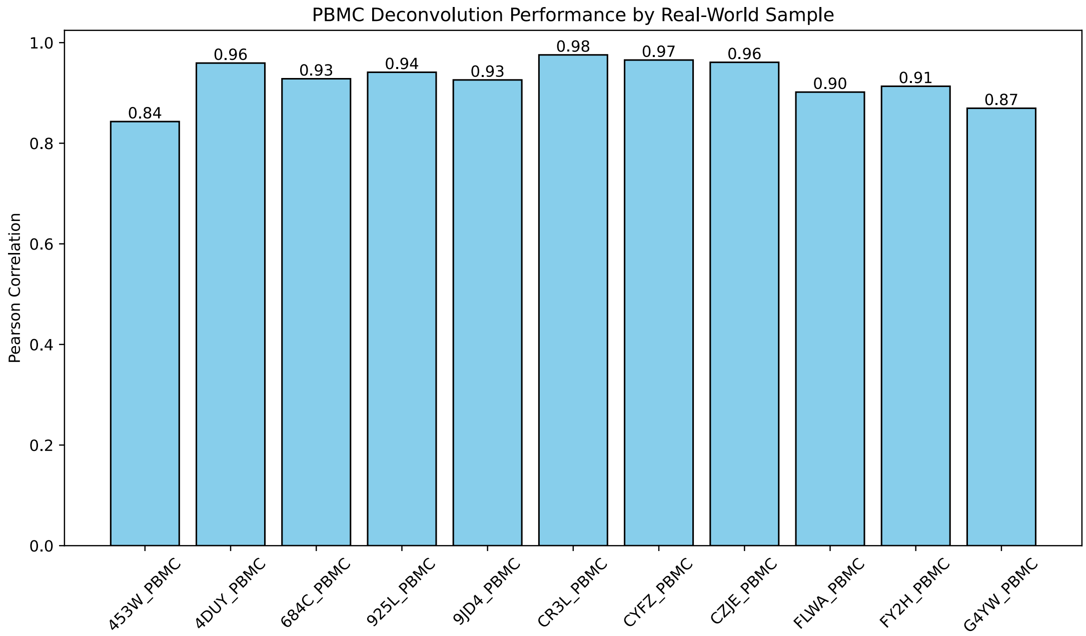

S12 Fig Validation of the CCD model on real PBMC bulk RNA-seq samples from GSE107011. Pearson correlation coefficients between model-inferred cell-type proportions and ground truth proportions derived from sorted PBMC populations.
