# Supplementary material for: LIMPACAT: Multi-omics attention transformer for immune prediction in liver cancer using whole-slide imaging
Source: PLoS One. 2026 Jan 9;21(1):e0339667. doi: 10.1371/journal.pone.0339667 (PMC12788640; doi:10.1371/journal.pone.0339667)
Supplement: S3 Fig — (A) shows the mt percent for each sample, reflecting cell viability and quality, with higher percentages indicating potential cellular stress or damage. (B) displays the nCount distribution, representing the sequencing depth across samples, which captures gene expression patterns specific to different cell types or states. (PDF) [file pone.0339667.s003.pdf]

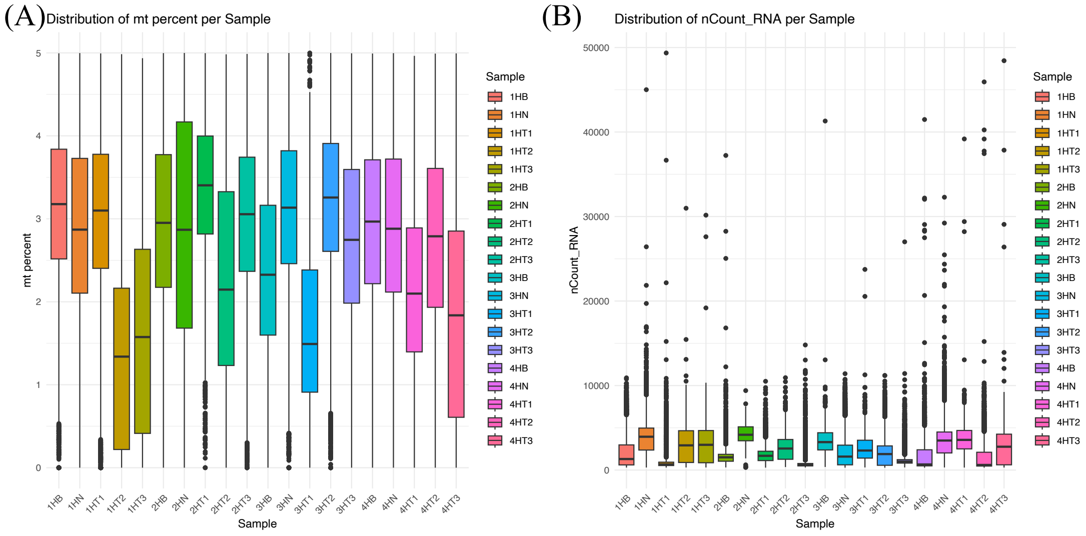

S3 Fig Distribution of mitochondrial gene percentage (mt percent) and nCount across samples post-filtering. (A) shows the mt percent for each sample, reflecting cell viability and quality, with higher percentages indicating potential cellular stress or damage. (B) displays the nCount distribution, representing the sequencing depth across samples, which captures gene expression patterns specific to different cell types or states.
