# Supplementary material for: LIMPACAT: Multi-omics attention transformer for immune prediction in liver cancer using whole-slide imaging
Source: PLoS One. 2026 Jan 9;21(1):e0339667. doi: 10.1371/journal.pone.0339667 (PMC12788640; doi:10.1371/journal.pone.0339667)
Supplement: Attachment — Submitted filename: Response.docx [file pone.0339667.s025.docx]

Journal Requirements:

1. Please ensure that your manuscript meets PLOS ONE's style requirements, including those for file naming. The PLOS ONE style templates can be found at

<https://journals.plos.org/plosone/s/file?id=wjVg/PLOSOne_formatting_sample_main_body.pdf>

and

[https://journals.plos.org/plosone/s/file?id=ba62/PLOSOne_formatting_sample_title_authors_affiliations.pdf](https://track.editorialmanager.com/CL0/https:%2F%2Fjournals.plos.org%2Fplosone%2Fs%2Ffile%3Fid=ba62%2FPLOSOne_formatting_sample_title_authors_affiliations.pdf/1/010f0198bb3d3e73-565e1251-41d5-4791-9e24-0868561e3437-000000/tDzAdk8bGzkcYI-W7CEAdToLcX8yRJIaQzFw7ArsZ94=224)

Response 1

We thank the editor for pointing this out. We have checked and revised the formatting. Author name and affiliation formatting will be adjusted according to the PLOS ONE style. Supplementary figure labels and in-text citations have been updated for consistency with the manuscript.

2. Please note that PLOS ONE has specific guidelines on code sharing for submissions in which author-generated code underpins the findings in the manuscript. In these cases, we expect all author-generated code to be made available without restrictions upon publication of the work. Please review our guidelines at <https://journals.plos.org/plosone/s/materials-and-software-sharing#loc-sharing-code> and ensure that your code is shared in a way that follows best practice and facilitates reproducibility and reuse.

Response 2

Software and data availability

We thank the editor for this reminder. The Software and Data Availability section has been revised. All custom code is openly available at GitHub (https://github.com/holiday01/LIMPACAT.git) and archived in Zenodo (10.5281/zenodo.17168482).

3. Thank you for stating the following in the Acknowledgments Section of your manuscript:

This work was supported by the grants from the National Science and Technology Council (NSTC).

We note that you have provided funding information that is not currently declared in your Funding Statement. However, funding information should not appear in the Acknowledgments section or other areas of your manuscript. We will only publish funding information present in the Funding Statement section of the online submission form.

Please remove any funding-related text from the manuscript and let us know how you would like to update your Funding Statement. Currently, your Funding Statement reads as follows:

This research was supported by grants from the National Science and Technology Council (NSTC), Taiwan (NSTC 112-2222-E-130-003- and NSTC 113-2221-E-130-005-MY3), which assisted in the acquisition of computational facilities essential for this study.

Please include your amended statements within your cover letter; we will change the online submission form on your behalf.

Response 3

We thank the editor for highlighting this. The Funding section has been revised to include full grant information.

4. Thank you for stating the following in your Competing Interests section:

No available

Please complete your Competing Interests on the online submission form to state any Competing Interests. If you have no competing interests, please state "The authors have declared that no competing interests exist.", as detailed online in our guide for authors at http://journals.plos.org/plosone/s/submit-now

This information should be included in your cover letter; we will change the online submission form on your behalf.

Response 4

We thank the editor for the clarification. The Competing Interests section has been revised to state that the authors declare no competing interests.

5. In the online submission form, you indicated that your data will be submitted to a repository upon acceptance. We strongly recommend all authors deposit their data before acceptance, as the process can be lengthy and hold up publication timelines. Please note that, though access restrictions are acceptable now, your entire minimal dataset will need to be made freely accessible if your manuscript is accepted for publication. This policy applies to all data except where public deposition would breach compliance with the protocol approved by your research ethics board. If you are unable to adhere to our open data policy, please kindly revise your statement to explain your reasoning and we will seek the editor's input on an exemption.

Response 5

We thank the editor for this note. All datasets generated in this study have been deposited and are openly accessible at GitHub (https://github.com/holiday01/LIMPACAT.git) and archived in Zenodo (10.5281/zenodo.17168482). These include derived datasets such as TCGA-LIHC WSI file identifiers, train/validation/test splits, and deconvolution labels. For external datasets, the raw WSIs are publicly available from the GDC Data Portal (https://portal.gdc.cancer.gov/), and the RNA-seq dataset is available under GEO accession, with download instructions provided by the respective repositories.

6. Please amend your list of authors on the manuscript to ensure that each author is linked to an affiliation. Authors’ affiliations should reflect the institution where the work was done (if authors moved subsequently, you can also list the new affiliation stating “current affiliation:….” as necessary).

Response 6

We thank the editor for the reminder. All author affiliations have been carefully checked and revised to ensure that each author is explicitly linked to an institution. Current affiliations have also been indicated where applicable.

7. Please upload a new copy of Figure 1 as the detail is not clear. Please follow the link for more information: <https://blogs.plos.org/plos/2019/06/looking-good-tips-for-creating-your-plos-figures-graphics/>"" [https://blogs.plos.org/plos/2019/06/looking-good-tips-for-creating-your-plos-figures-graphics/](https://track.editorialmanager.com/CL0/https:%2F%2Fblogs.plos.org%2Fplos%2F2019%2F06%2Flooking-good-tips-for-creating-your-plos-figures-graphics%2F/1/010f0198bb3d3e73-565e1251-41d5-4791-9e24-0868561e3437-000000/v73UBwv-L3zizUZDuODxBC6TrB5_Y4G8PweBocgNLg8=224)

Response 7

We thank the editor for this important reminder. All supplementary figures and tables have been consolidated into a single file. Each element is labeled according to PLOS ONE style (e.g., S1 Fig, S2 Fig, S1 Table). In-text citations have been revised accordingly.

8. Please include captions for your Supporting Information files at the end of your manuscript, and update any in-text citations to match accordingly. Please see our Supporting Information guidelines for more information: <http://journals.plos.org/plosone/s/supporting-information>.

Response 8

We thank the editor for this important reminder. All supplementary figures and tables have been consolidated into a single file (“S1 File. Supplemental Figures and Tables”). Each element is labeled according to PLOS ONE style (e.g., S1 Fig, S2 Fig, S1 Table). In-text citations have been revised accordingly.

9. If the reviewer comments include a recommendation to cite specific previously published works, please review and evaluate these publications to determine whether they are relevant and should be cited. There is no requirement to cite these works unless the editor has indicated otherwise.

Response 9

We thank the editor for this note. We have reviewed the suggested references. Relevant publications will be cited where appropriate, while others will be carefully evaluated in the context of our study.

Reviewer #1

1. Throughout the manuscript the authors refer to “liver cancer” or “LIHC” without specifying that the study is restricted to HCC. Because intrahepatic cholangiocarcinoma and combined HCC-ICC have distinct immune microenvironments.

Reviewer #1Response1

We appreciate the reviewer for this valuable comment. We have revised the manuscript to clarify that all analyses are restricted to hepatocellular carcinoma (HCC). Ambiguous terms such as “liver cancer” have been replaced with “HCC” throughout the revised manuscript. We explicitly indicate that the GSE189903 dataset comprises 20 HCC samples and clarify that the TCGA-LIHC cohort, as defined by The Cancer Genome Atlas, includes only HCC cases, excluding intrahepatic cholangiocarcinoma (ICC) and combined HCC-ICC cases. (Line 136-139)

2. The Abstract and Discussion repeatedly label the approach as “non-invasive.” Since WSIs are derived from surgically resected or biopsied specimens, the technique is technically post-operative/ex vivo, not non-invasive.

Reviewer #1Response 2

We appreciate the reviewer for this important clarification. We agree that whole-slide images (WSIs) are derived from surgically resected or biopsied tissues and, therefore, the approach should not be described as “non-invasive.” Consequently, we have revised the Abstract and Discussion to more precisely state that our method enables *ex vivo* immune profiling and pathology-based tumor microenvironment characterization. (Line 12)

3. The study relies solely on publicly available TCGA-LIHC WSIs and GEO scRNA-seq data. The manuscript currently lacks independent clinical validation (e.g., a local HCC cohort with flow-cytometry or multiplex-IHC ground truth). Please add prospective validation with HCC specimens.

Reviewer #1 Response 3

We appreciate the reviewer for highlighting this important point. We acknowledge that the lack of prospective validation using a local HCC cohort with flow cytometry or multiplex immunohistochemistry (mIHC) reference data constitutes a limitation of this study. To address this concern, we implemented a two-step validation strategy. First, we used HCC single-cell RNA sequencing (scRNA-seq) data to generate simulated bulk RNA-seq samples, demonstrating the feasibility of the cell composition deconvolution (CCD) framework. (Line 403-424)

Second, in this revision, we added to perform independent validation using newly generated peripheral blood mononuclear cell (PBMC) bulk RNA-seq data with experimentally defined immune cell populations. This additional dataset confirmed the robustness of the CCD model beyond simulated data (Line 422-424).

4. Figure 5A (UMAP after log-normalization) is supplied at insufficient resolution. Please provide a high-resolution vector graphic.

Reviewer #1Response 4

We thank the reviewer for the comment. Figure 5A (UMAP after log-normalization) has been revised and is now provided as a high-resolution vector graphic.

5. Report exact p-values (not “p < 0.05”) for all log-rank tests in Supplementary Figure 10E–H and include 95 % confidence intervals for the accuracy metrics in Table 1.

Reviewer #1Response 5

We appreciate the reviewer for this insightful suggestion. Ninety-five percent confidence intervals for all accuracy metrics in Table 1 have been included, calculated from the sample mean and standard error based on 30 repeated model training runs using train-validation splits. Additionally, the exact p-values for all log-rank tests have now been reported. Owing to resolution issues, the corresponding plots have been reformatted and are now presented in S11 Fig.

6. Provide a brief justification for selecting log-normalization over CCA or SCTransform, despite the latter showing tighter batch correction.

Reviewer #1Response 6

Thank you for your valuable comment. As outlined in the Results section under “Cell Composition Deconvolution,” we compared three normalization methods: CCA, SCT, and log normalization. Log normalization demonstrated the highest clustering consistency, as measured using the adjusted Rand index (ARI), and preserved greater biological variability across samples, which is essential for capturing tumor microenvironment heterogeneity. These findings, consistent with those of ARI metrics and correlation analyses (S10 Fig), informed our decision to use the log-normalized CCD model for downstream survival analyses. (Line 343-355)

Reviewer #2

1- Technical validity and data support:

The manuscript is partially supported by the data. While the use of deconvolution from scRNA-seq to simulate bulk RNA-seq is methodologically reasonable, there is no direct experimental validation of the immune cell predictions (e.g., via flow cytometry, IHC, or spatial transcriptomics). Moreover, all model training and evaluation were conducted using a single dataset (TCGA-LIHC), limiting generalizability.

Reviewer #2 Response 1

We acknowledge that our study does not include direct experimental validation, such as flow cytometry, IHC, or spatial transcriptomics, which would provide the most definitive confirmation of immune cell proportions.

However, beyond using simulated bulk RNA-seq generated from scRNA-seq—a widely adopted proxy supported by previous studies demonstrating reasonable concordance with experimental measurements—we conducted external validation using real-world PBMC bulk RNA-seq data from the GSE107011 dataset. This dataset comprises bulk RNA-seq profiles of sorted immune cell populations with well-characterized compositions, enabling us to evaluate model performance against experimentally defined ground truth. The comparison demonstrated strong concordance, quantified using Pearson correlation coefficients (PCCs), thereby supporting the robustness of our deconvolution model in real bulk RNA-seq contexts. (Line 251-255, 419-427)

We have revised the Discussion to highlight both the absence of direct experimental validation in liver cancer tissues and the inclusion of PBMC-based validation, partially mitigating this limitation. (Line 526-540)

2 -Statistical analysis:

Statistical approaches are broadly appropriate but lack sufficient rigor and transparency. There is no report of confidence intervals or statistical significance for model accuracy, nor of hazard ratios in survival analyses. No corrections for multiple hypothesis testing are mentioned, and data splitting or cross-validation strategies for model training are not described, raising concerns about potential overfitting.

Reviewer#2Response 2

We appreciate the reviewer for this valuable suggestion. To enhance the statistical transparency of our study, we have clarified the evaluation metrics used for each analysis. For the deconvolution model, performance was assessed using the PCC, a standard commonly used to benchmark cell composition predictions. Additionally, we present results derived from both simulated and real bulk RNA-seq datasets (GSE107011). (Line 251-255, 419-427)

For the WSI-based classification analyses, we have added 95% confidence intervals to all accuracy metrics in Table 1, calculated from the sample mean and standard error across 30 repeated training runs with train–validation splits. For the survival analyses, the exact p-values of all log-rank tests are now reported. Corresponding plots have been reformatted and moved to S11 Fig. for improved clarity. These revisions enhance the rigor and transparency of our statistical reporting. (Line 262-266)

3- Language and presentation:

The manuscript is not yet written in fully standard English. While the technical content is generally understandable, there are multiple grammatical and syntactic issues that require revision. Additionally, some sections (especially Methods and Discussion) are repetitive or overly verbose. A professional language edit is strongly recommended to improve clarity and readability.

In summary, this manuscript presents a creative and impactful contribution to digital pathology and computational immuno-oncology. With improved statistical rigor, clearer methodological reporting, and stronger language polishing, it has the potential to make a meaningful contribution to the field.

Reviewer#2Response 3

We appreciate the reviewer’s comment regarding the language quality. The manuscript has already undergone professional English editing, and the revised version with proof of editing has been uploaded with this submission.

Reviewer #3

Major Comments:

1. Clarity of Model Architecture and Workflow:

The inputs and outputs of the MIL-ATTENTION model are unclear in Figure 1 and throughout the manuscript. What is the output of the MIL-ATTENTION model? Figure 1 should also clearly distinguish between the training pipeline and the inference pipeline for both the ensemble DL-CCD model and the MIL-ATTENTION model, including the type of data and processing applied in each.

Reviewer #3Response1

We appreciate the reviewer for this valuable comment. In response, we have revised both the text and Fig 1 to more clearly illustrate the architecture and workflow of the MIL-ATTENTION model and its integration with the ensemble DL-CCD framework. Specifically, we have updated Figure 1 to distinctly separate the training pipeline (including scRNA-seq–derived pseudo-bulk RNA-seq for deconvolution, generation of immune infiltration groupings, and supervision of WSI-based MIL training) from the inference pipeline (involving applying the trained DL-CCD model to real bulk RNA-seq and the MIL-ATTENTION model to WSIs). The updated figure now clearly indicates the data type used at each stage (scRNA-seq, simulated bulk RNA-seq, real bulk RNA-seq, WSIs) along with the corresponding processing steps. (Line 106-125)

2. Positioning Against Existing Methods:

Many deconvolution models exist for bulk RNA-seq. The authors need to clearly articulate the advantages of their DL-CCD model over established methods.

Reviewer #3Response2

We appreciate the reviewer for this valuable comment. To clarify the positioning of our model, we have revised the Introduction to explicitly compare DL-CCD with established bulk RNA-seq deconvolution methods, such as quanTIseq and CIBERSORTx. Specifically, we emphasized that DL-CCD builds upon our previously developed HASCAD framework, validated using real bulk RNA-seq data from PBMCs and purified immune cell populations. (Line54-56 and Line 203-207)

In this revision, we further incorporated PBMC data GSE107011 for testing and confirmed that the model achieves high accuracy. (Line 251-255, 419-427)

3. Reorganization of the Results Section:

The sections from “Gene Count and Quality Control Metrics” to “Single-Cell Type Annotation” are too detailed for the Results section and should be condensed into a brief summary, with the full methodological descriptions moved to the Methods section. The Results should highlight findings that are novel and relevant to the field.

Reviewer #3Response3

We appreciate the reviewer for this observation. To improve clarity, we have revised the Results section to more clearly describe the interaction between the ensemble-DNN deconvolution model and the WSI classifiers in Figure 1. (Line 106-125)

Specifically, we added a bridging paragraph explaining that predicted immune proportions were used to stratify LIHC samples into high- and low-infiltration groups, subsequently serving as pseudo-labels for training the WSI classifiers. (Line270-276)

This revision clarifies the interplay between the components and highlights how the framework can be adopted to other datasets.

4. Validation Using Real Bulk RNA-seq Data:

The evaluation of the cell deconvolution model appears to rely primarily on simulated bulk RNA-seq derived from scRNA-seq. However, scRNA-seq and actual bulk RNA-seq have distinct characteristics. The model should be validated on real-world bulk RNA-seq datasets with known or estimated cell compositions to establish robustness.

Reviewer #3Response4

Thank you for your valuable suggestion. In response, we have performed additional validation using real-world bulk RNA-seq data from the GSE107011 dataset, comprising PBMC samples derived from sorted immune cell populations. This dataset provides biologically relevant ground truth for evaluating the accuracy of the deconvolution model. (Line 251-255)

To evaluate the robustness of the model, we applied the CCD model to these bulk RNA-seq samples. We compared the inferred cell-type proportions with the expected values using the PCC, a standard metric in cell deconvolution benchmarking. The model exhibited consistently high correlations across samples (r = 0.84–0.98), indicating strong concordance with the ground truth compositions. (Line 419-427)

5. Clarity and Framework Usability:

The section titled “Cell Composition Deconvolution Model and Immune Cell Level Estimated from WSI” is currently difficult to follow and lacks detail. It is the core of the paper and should be rewritten to clearly describe how the model components interact. It should also contain information about how the overall framework can be used by others.

Reviewer #3Response5

Thank you for the valuable suggestion. We have revised the “Immune Cell Prediction Model Development Using scRNA-seq” and “Digital Pathology Image Analysis” sections to clarify the components of the model and their interactions. Additionally, we have included a summary of the entire workflow in the Study Design section to enhance clarity and reproducibility. (Line 106-125)

6. Overfitting and Generalization:

There appears to be overfitting across all cell types. The authors should address this issue and describe any strategies used to mitigate overfitting (e.g., regularization, validation, dropout, etc.).

Reviewer #3Response6

We thank the reviewer for pointing out the concern regarding potential overfitting. In our study, we employed several strategies to mitigate overfitting, including data augmentation of WSI tiles (random flips and rotations), dropout layers in the MIL models, weight decay regularization, and early stopping based on validation performance. We also report results with 95% confidence intervals to emphasize model robustness. Furthermore, the consistency between CCD-based predictions and WSI-based predictions across independent cohorts supports the generalizability of our approach. (Line 430-440)

Minor Comments:

7. Acronyms such as “WSIs” and “CCD” should be spelled out at first mention. This is especially important in the abstract and background sections.

Reviewer #3Response 7

We appreciate the reviewer for this insightful suggestion. We have revised the manuscript to spell out “whole-slide images (WSIs)” and “cell composition deconvolution (CCD)” at their first mention, including in the Abstract and Background sections.

8. Several sentences in the Background section (e.g. paragraphs 2 and 3) lack citations.

Reviewer #3Response8

Thank you for highlighting this. We have revised the Background section to include appropriate citations for the statements in paragraphs 2 and 3.

9. Why using CCA and SCT normalization on LIHC bulk RNA-seq samples?

Reviewer #3Response 9

We appreciate the reviewer for highlighting this. To clarify, CCA and SCT normalization were applied exclusively to the scRNA-seq data during preprocessing to correct for batch effects across samples. (343-355)

In contrast, the bulk RNA-seq expression profiles from TCGA-LIHC were processed using upper-quartile normalization, as described in the Methods. (Line 164)

Reviewer #4

1. The methodology section is short and several important methodological steps are only introduced in the results. The first paragraphs of the results section do not contain any results and instead focus on quality control steps, software libraries and clustering methods. A clearer separation of methods and results would improve readability.

Reviewer #4Response 1

We appreciate the reviewer for this feedback. In the revised manuscript, we have substantially expanded the Methods section to include details previously presented in the Results section. Specifically, descriptions of quality control steps, software libraries, and clustering methods were moved to the Methods section. The Results section now begins directly with the presentation of results, providing a clearer separation between methodology and findings.

2. The data sets used are insufficiently characterized. I could not find information on the survival analysis data set, including number of patients that had matched WSIs and survival data available, their demographic and disease characteristics, the duration of follow up and whether there was any clustering present that could influence the statistical analysis (is there one slide per patient or potentially multiple?).

Reviewer #4Response 2

We thank the reviewer for highlighting this observation. In this study, all tumor samples were derived from HCC cases within the TCGA-LIHC dataset. The TCGA histological annotations include several subtypes, such as hepatocellular carcinoma (not otherwise specified, NOS), clear cell type, fibrolamellar, spindle cell variant, and combined HCC-cholangiocarcinoma. Per TCGA classification, these are categorized as HCC or HCC-related variants; therefore, all cases in our analysis represent HCC. To ensure clarity, we have revised the Methods section to explicitly list these histological subtypes and to confirm that neither intrahepatic cholangiocarcinoma nor other primary liver cancer types were included. (Line 165-172)

3. It is not entirely clear why only 20 samples were chosen from the GSE189903 dataset. This is generally a very small sample. Were the other 14 from different types of liver cancer (that would be a very high proportion) or was this number based on statistical or computational considerations? Do the 20 samples correspond to 20 different patients?

Reviewer #4Response 3

We appreciate the reviewer for this insightful comment. The GSE189903 dataset comprises 34 primary liver cancer samples, including both HCC and iCCA. Since our study focused specifically on HCC, we included only the 20 HCC samples, corresponding to patients in the TCGA-LIHC cohort. This approach ensures disease specificity and avoids potential confounding from other liver cancer subtypes. We have clarified this in the Methods – Data Download and Preprocessing section. (Line 136-143)

4. The validation using survival analysis is insufficiently described and could be more thorough. It is not clear how low and high CCD prediction groups were determined, where the cutoff was set and why CCD was not validated as a continuous predictor. Likewise, the paper mentions good and poor survival groups, but does not describe how they were determined. Using the raw prediction scores and survival times directly for validation would have been better. Instead of accuracy the C-index could have been reported. The method for splitting the sample into train/test or cross validation folds is not described. Uncertainty should be quantified. Alternative explanations for the reported associations should at least be discussed. A multivariate model adjusted for (at least) tumor stage could improve interpretability.

Reviewer #4Response 4

We appreciate the reviewer for this valuable feedback. In this study, survival analyses were conducted using the Kaplan–Meier method, stratifying patients into high- and low-risk groups based on the median immune cell fractions predicted using our model. The median cutoff was chosen to align with the weak supervision strategy used during model training, which utilizes binary scRNA-seq–derived labels. In addition, we also performed survival analyses using immune levels predicted directly from WSIs, and the results were consistent with those obtained from the CCD-based predictions. (Line 442-445)

While we acknowledge that using continuous immune scores and reporting the concordance index (C-index) would offer a more granular evaluation, our primary focus was to demonstrate the feasibility and interpretability of weakly supervised immune inference from WSIs. We have clarified these methodological decisions and their limitations in the Methods and Discussion sections, recommending that future studies incorporate multivariate models with continuous predictors and relevant clinical covariates. (Line 548-558)

Reviewer #5

Major Revisions
1.Validation of Deconvolution Predictions: The most significant limitation of this study is the reliance on computationally inferred immune cell compositions as ground truth labels without subsequent biological validation. The model's high accuracy indicates it can successfully predict these inferred labels, but whether these labels accurately reflect the true cellular makeup of the TME is unknown. The authors should:
① More explicitly and prominently state this limitation in the Discussion and Conclusion sections.

Reviewer #5Response 1-1

We appreciate the reviewer for this valuable suggestion. We have revised both the Discussion and Conclusion sections to explicitly acknowledge the limitation of lacking direct biological validation for the predicted immune cell abundances. Specifically, we have now emphasized that while our study demonstrates the feasibility of integrating scRNA-seq and WSI data for immune cell prediction, the absence of experimental validation methods, such as IHC or multiplex immunofluorescence (mIF), remains a key limitation. Additionally, we highlighted that future studies should incorporate these validation strategies to confirm the biological accuracy of our predictions. (Line 541-547)

② Suggest future work involving validation against a ground truth, such as performing immunohistochemistry (IHC) or multiplex immunofluorescence (mIF) for the predicted cell types (B cells, NK cells) on a subset of the WSI slides to correlate cell counts. For an example of how such validation is performed, the authors could refer to recent studies that correlate computational predictions with IHC.

Reviewer #5Response 1-2

We appreciate the reviewer for this valuable suggestion. We validated our framework using real bulk RNA-seq data from PBMCs and purified immune cell populations, which provides supportive evidence for the accuracy of our predictions. (Line 419-427)

2. Comparison with State-of-the-Art: The discussion compares LIMPACAT to several other models, including CLAM. However, the field of transformers in computational pathology is advancing rapidly. The manuscript would be significantly strengthened by discussing and contextualizing LIMPACAT with other very recent (2023-2025) transformer-based architectures for WSI analysis and immune prediction. This would provide a clearer picture of where LIMPACAT stands in the current landscape.

Reviewer #5Response 2
We appreciate the reviewer for this valuable suggestion. In the revised Discussion, we have added a dedicated section positioning LIMPACAT within the context of recent transformer-based approaches. Specifically, we discussed foundation models (UNI, Virchow, Prov-GigaPath) demonstrating the potential of large-scale self-supervised encoders; advanced multiple instance learning (MIL) aggregators such as HVTSurv, H-TransMIL, and MambaMIL prioritizing sequence-efficient and multi-scale modeling; and tumor microenvironment–focused methods such as HistoTME connecting WSI-derived immune estimates to clinical outcomes. In contrast, LIMPACAT integrates single-cell–derived deconvolution to generate biologically grounded immune labels for weak supervision. This approach complements foundation model pretraining and advanced MIL architectures, positioning LIMPACAT as a conceptual bridge between single-cell biology and transformer-based computational pathology, with clear potential for future integration and extension. (Line 510-525)

Minor Revisions
1. Improvement of figure quality:
The images of Supplementary Figure 10 (A)-(E) were exported out rather low resolution. Please ensure image clarity.

Reviewer #5Response 1

We appreciate the reviewer for this helpful feedback. In the original layout, S10A–E Fig. presented all subfigures within a single panel, resulting in crowding and reduced clarity. To improve presentation, we have split the content into two separate figures (S10 & S11 Fig.), each with higher resolution and an optimized layout to enhance readability and image quality. The updated figures have been included in the revised supplementary materials.

2.Methodological Clarity:
①In the Methods section, please specify the exact threshold or method used to stratify patients into "high" and "low" immune cell groups for the survival analysis (e.g., median, quartiles).

Reviewer #5Response 2-1

We appreciate the reviewer for this constructive suggestion. In the revised Methods section, we have specified that, in the log-rank survival analysis, patients were stratified into “high” and “low” immune cell groups based on the median estimated immune cell fraction. This stratification was used to identify immune cell types with significant prognostic differences. (Line 270-276)

②Provide a more detailed description of the custom CNN, ATT, and ATT_TRANS model architectures, either in the main text or as a supplementary methods section.

Reviewer #5Response 2-2

We appreciate the reviewer for this helpful suggestion to clarify the preprocessing and model design. We have revised the Methods section to provide a more detailed description of the WSI patching strategy, quality control procedures, and the three MIL-based model architectures. (Line 214-249)
