# Supplementary material for: LIMPACAT: Multi-omics attention transformer for immune prediction in liver cancer using whole-slide imaging
Source: PLoS One. 2026 Jan 9;21(1):e0339667. doi: 10.1371/journal.pone.0339667 (PMC12788640; doi:10.1371/journal.pone.0339667)
Supplement: Attachment — Submitted filename: Response to Reviewers.docx [file pone.0339667.s026.docx]

Reviewer #1: The authors have addressed all of my concerns in a detailed and rigorous manner. They have made the necessary revisions to the manuscript, and I believe that the current version of the article is now acceptable for publication.

Response Reviewer #1:

We thank the reviewer for the positive evaluation and for acknowledging the improvements made in the revised manuscript.

Reviewer #3: Many places in the manuscript contain "Error! Reference source not found" that should be fixed.

Response Reviewer #3:

We thank the reviewer for identifying this issue. All cross-reference and citation errors have been carefully reviewed and fully corrected throughout the revised manuscript. No remaining “Error! Reference source not found” messages appear in the updated version.

Re Reviewer #3Response2, please point out the paragraph that "explicitly compare DL-CCD with established bulk RNA-seq deconvolution methods, such as quanTIseq and CIBERSORTx".

Response Re Reviewer #3Response2:

We thank the reviewer for the comment. The previous discussion referred to comparisons with conventional methods such as quanTIseq and CIBERSORTx through cited studies, but this was not explicitly stated. We have now added a clear sentence in the Introduction to explicitly indicate these methods.

The following text has been added in the Introduction (Lines 51–58):

“Early CCD models relied on regression model frameworks such as quanTIseq and CIBERSORTx, which depend on predefined immune signatures and therefore struggle to capture nonlinear gene–cell relationships and tumor-specific immune heterogeneity. More recent studies have demonstrated that incorporating single-cell RNA sequencing (scRNA-seq) references with deep learning (DL) architectures substantially improves deconvolution accuracy. In our previous work, we introduced a DL-based CCD model that achieved higher concordance with true immune cell fractions than both CIBERSORTx and quanTIseq [8].”

Reviewer #4: My comments were adequately addressed. I support acceptance given the additional inclusion of a separate validation cohort.

Response Reviewer #4:

We sincerely thank the reviewer for the positive feedback and for recognizing the improvements made in the revision. We appreciate the reviewer’s earlier comments, which helped strengthen our validation strategy and clarify the contribution of our method.
